# Supplementary material for: Are carbon emissions trading and green financial instruments synergistic? -Comprehensive quantitative research based on content analysis
Source: PLoS One. 2024 Mar 7;19(3):e0298601. doi: 10.1371/journal.pone.0298601 (PMC10919601; doi:10.1371/journal.pone.0298601)
Supplement: S1 Data — (ZIP) [file pone.0298601.s001.zip › Data/地级市Z_F工作报告.zip!/╒π╜¡/║■╓▌╩╨/└┤╫╘╣1⁄2╓┌║┼í╛┬φ┐╦╩2╛▌═°í┐_2014.docx]

各位代表：

　　现在，我代表市人民政府，向大会作工作报告，请予审议，并请市政协委员和其他列席人员提出意见。

　　一、2013年主要工作

　　过去的一年，面对错综复杂的宏观经济环境和历史罕见的重大自然灾害，全市上下认真贯彻党的十八大和十八届二中、三中全会精神，牢牢把握“稳中求进、转中求好”总基调，深入实施“八大专项行动”，扎实开展“两看两比”活动，现代化生态型滨湖大城市建设取得新的成就。地区生产总值达到1803.2亿元，比上年增长9%；固定资产投资1070亿元，增长10.2%；社会消费品零售总额766.2亿元，增长13.1%；外贸进出口总额95.3亿美元，其中出口80.9亿美元，分别增长9.3%和9.5%；财政总收入271.7亿元，其中地方财政收入154.7亿元，分别增长10%和11.6%；城镇居民人均可支配收入36220元，农村居民人均纯收入19044元，分别增长9.8%和10.8%；城镇新增就业5.82万人，登记失业率3.07%；居民消费价格总水平上涨2.1%；人口自然增长率2.2‰。预计研究与试验发展经费支出占生产总值比例达到2.3%，单位生产总值能耗下降3.5%，化学需氧量、氨氮、二氧化硫、氮氧化物排放量分别削减2.8%、 2.8%、 3.5%和4%以上。除固定资产投资等个别指标外，市七届人大三次会议确定的主要预期目标顺利实现；十方面民生实事项目全面完成；宁杭高铁建成通车，我市跨入“高铁时代”；争创国家生态文明建设先行示范区取得积极进展，成功创建国家森林城市，国家历史文化名城申报工作通过专家组考察评估。

　　（一）经济转型升级扎实推进。以有效投入促进经济增长。统筹浙商回归、外资利用、央企合作，推动招商引资聚焦“大好高”项目，到位省外浙商回归资金144.6亿元、外资10.6亿美元，新签约总投资10亿元以上项目16个。开展“百个项目、百日攻坚”行动，市重点建设完成投资474.6亿元，工业“双十双百”、服务业“双百”计划均超额完成。提升产业平台集聚功能，南太湖产业集聚区优化提升、省际承接产业转移示范区开发建设扎实推进。加强重大项目用地保障，新增建设用地指标2.4万亩，盘活存量建设用地5129亩，批而未供土地净减少4352亩。安吉获得“全国国土资源节约集约模范县”称号。强化金融支持实体经济，新增金融机构贷款231.5亿元，完成直接融资121.4亿元，小额贷款公司等新型金融组织稳步发展，民间融资规范管理试点扎实推进。

　　以工业强市引领产业升级。加快推进工业结构调整，六大重点特色产业增加值占比提高到52.1%，高新技术产业、战略性新兴产业、装备制造业增加值分别增长12%、11.8%和14.4%，南浔电梯等一批特色产业快速发展。印染、造纸、制革、化工四大行业列入淘汰计划的76家企业全部关停到位，吴兴区织里童装产业转型升级取得积极进展，长兴县成功打造“全国粉体产业转型升级示范区”。出台加快服务业发展“20条”，服务业增加值占比提高到40.2%。农贸市场改造提升三年行动计划全面完成，湖州文化发展集团、旅游发展集团相继成立，安吉县成为国家乡村旅游度假实验区，浙江省长三角金融后台基地落户德清。大力发展品质农业，新建成粮食生产功能区10.4万亩、现代农业园区8.3万亩，农技研发与推广体制机制创新改革试点扎实推进，湖州农业科技园区晋升“国字号”，吴兴区被列为国家现代农业示范区农业改革与建设试点。出台支持开放型经济发展“20条”，外贸出口稳定增长，服务外包产业快速发展。

　　以创新驱动增强发展活力。出台创新驱动发展“22条”，推进科技创新“六个一”重点工作，我市入围国家创新型试点城市和知识产权示范城市。加强创新平台建设，省级现代物流装备高新技术产业园区挂牌设立，德清省级科技成果转化实验区加快建设，长兴国家大学科技城、安吉科教文新区建设全面推进。强化政产学研金合作，与中科院、浙江大学等大院名校的合作不断深化，市本级首家科技银行组建成立。制定优化企业人才服务“10条”，新引进“南太湖精英计划”领军人才79名，新入选“国千” 6人、“省千” 9人。大力推动企业建立研发机构、提升创新能力，新认定国家重点扶持高新技术企业62家。实施“四换三名”工程，在创新发展中培大育强、扶小促优，纳税超千万元企业新增51家，达到370家，其中超亿元企业18家；完成“个转企” 6060家、“规下转规上” 229家、“限下转限上” 252家。

　　（二）城乡环境面貌持续改善。城市功能品位不断提升。统筹交通等重大基础设施建设，杭长高速公路北延项目正式开工， 318国道改线工程加快实施，长湖申航道浙江段扩建工程全线建成。有序推进湖州中心城市重点城建项目，梁希森林公园一期建成开放， 10个老小区综合整治全面完成，新浙北、东吴国际相继落成，外环线、奥体中心等建设步伐加快。整体推进中心城市建设，湖州开发区综合交通枢纽区块框架全面拉开，太湖度假区滨湖开发建设水平不断提升，吴兴东部新区建设持续推进，南浔城区功能形象切实改善。全面治理城市交通拥堵，打通了一批断头路，改善了一批拥堵点，全市投放公共自行车5550辆。中心城区集中整治百日行动取得实效，“数字城管”系统建成运行。三县城市建设力度进一步加大，织里镇、新市镇小城市培育试点不断深化，中心镇发展改革加速推进。

　　美丽乡村建设继续深化。新建成市级美丽乡村94个，安吉成为全国首个美丽乡村标准化创建示范县，德清被评为全省美丽乡村创建先进县。中心村建设加快推进，历史文化村落保护和利用工作进一步加强。完成农村土地综合整治复垦1.7万亩。市区美丽乡村路建设行动启动实施，全市农田水利五项标准化工程和气象现代化建设加快推进，农用电力线路改造工作全面开展。新创建五星级农村社区服务中心90个。大力推进新农村建设综合配套改革，八里店南片新农村综合改革试验区建设不断深化，南浔城南城乡一体化发展试验区建设全面启动，全市村级集体资产股份合作制改革完成率达到53.7%。

　　城乡环境整治扎实推进。完成旧住宅区、旧厂区、城中村改造1844.6万平方米，拆除违法建筑851.4万平方米，“三改一拆”实现“三年任务一年完成”。全面开展“四边三化”和“清理河道、清洁乡村”行动，一批环境“脏、乱、差”问题得到集中整治。组织实施河道清淤疏浚保洁等治水十大工程，全市河道逐一落实“河长制”，水环境治理工作基本实现“近期洁”的目标。成功列入全国首批水生态文明城市建设试点，四大重点水利工程全部获批立项。在全省率先开展大气复合污染防治工作，建筑扬尘、机动车尾气治理等十大工程扎实推进。铁腕推进矿山企业综合治理，拆除无矿山加工机组253套，建筑石料出港外销量压缩40%以上。加大农业面源污染治理力度，实施生猪、温室龟鳖养殖“双控”管理。全面推进生态创建工作，顺利通过省级生态市技术核查。

　　（三）社会民生事业加快发展。民生保障持续强化。实施高校毕业生创业引领计划，推进失业预警试点工作，就业形势保持稳定。社会保障覆盖面进一步扩大，全社会养老、医疗、失业、低保待遇水平得到新提高，在全省率先开展覆盖城镇职工、城乡居民的大病保险试点。开工建设城镇保障房项目46个，完成农村困难群众危旧房改造1111户。教育现代化建设加快推进，发展学前教育三年行动计划全面完成，政府助学工程深入实施。加强公共文化服务体系建设，建成农村文化礼堂116个，“文化走亲”项目荣获第十六届全国“群星奖”，文化发展指数提升至全省第三位。群众体育蓬勃发展，环太湖国际公路自行车赛等重大赛事成功举办。实施县级公立医院综合改革，完成103家社区卫生服务机构标准化建设，县级以上医院“一号通”系统建成使用。联合国人口基金“第七周期”项目扎实推进，“幸福家庭”促进计划深入实施。残疾人以及慈善、红十字等事业取得新进步。

　　社会建设不断加强。实施社会管理重点项目建设三年行动，完善重大决策社会稳定风险评估机制，开展“排查整治、强基促稳”专项行动，“平安湖州”建设取得新成绩。“六五”普法深入推进，法律援助不断加强。继续开展公安惠民十大行动，民意导向型警务模式创新发展。德清户籍管理制度改革试点稳步实施、取得实效。流动人口、特殊人群服务管理工作进一步加强。推进企业安全生产标准化和诚信管理机制建设，开展食品安全“强网清源”行动，公共安全得到有效保障。双拥工作以及国防后备力量建设不断深化，对口支援以及民族、宗教、外事、侨务、对台等工作取得新成绩。特别是有效应对人感染H7N9禽流感疫情和持续高温干旱、强台风“菲特”等重大自然灾害，把损失降到了最低。

　　（四）政府自身建设切实加强。自觉接受人大、政协等各方面的监督，人大代表建议、政协提案办理质量进一步提高。深化行政审批制度改革，新取消市级审批事项19项、下放83项、合并36项，审批服务效能进一步提高。整合建立“12345政府阳光热线”，全力打造“24小时不下班的政府”。严格执行中央“八项规定”和省、市办法，建立影响机关工作作风和效能行为问责制度，初步测算市级“三公”经费支出下降12.6%。加强行政监察和审计监督，严肃查处违纪违法案件，进一步规范公共资源交易，惩治和预防腐败体系建设深入推进。

　　各位代表！一年来的成绩来之不易。这是省委、省政府和市委正确领导的结果，是市人大、市政协以及各方面监督支持的结果，是全市人民克难攻坚、顽强拼搏的结果。在此，我代表市人民政府，向全市人民、外来建设者，表示衷心的感谢！向人大代表、政协委员，各民主党派、工商联、人民团体，以及离退休老同志、社会各界人士，表示衷心的感谢！向驻湖人民解放军和武警部队官兵，表示衷心的感谢！向所有关心和支持湖州发展的海内外朋友们，表示衷心的感谢！

　　我们也清醒地看到，发展中面临不少矛盾和困难，工作中存在一些差距和不足，主要是：市场需求明显减弱，一些行业产能过剩，少数企业困难加剧；有效投资增长乏力，特别是“大好高”项目不多，经济发展后劲不足；经济结构性、素质性矛盾突出，传统产业改造提升和新兴产业培育发展的任务十分艰巨；节能减排形势严峻，治水、治气和生态文明建设任重道远；民生改善离群众期望还有差距，影响社会和谐稳定的因素依然不少；政府自身改革步伐亟待加快，转变职能、提升效能以及加强政风建设都需要不断向纵深推进。我们有坚强的决心，有必胜的信念，一桩桩、一件件地克服困难、解决问题，努力把各项工作做得更好！

二、2014年工作总体要求

　　今年是贯彻落实党的十八届三中全会精神、全面深化改革的开局之年。改革红利不断释放，特色发展潜力巨大，机遇面前必须乘势而上。经济下行压力不减，区域竞争更为激烈，挑战面前必须迎难而上。我们一定要高举改革这面大旗，紧扣发展第一要务，用足用好区位、交通、生态、人文组合优势，以坐不住、等不起、慢不得的责任感和紧迫感，更加扎实有为地做好政府工作，向全市人民交出满意的答卷。

　　按照市委七届五次全会暨全市经济工作会议作出的部署，今年政府工作的总体要求是：以邓小平理论、“三个代表”重要思想、科学发展观为指导，全面贯彻落实中央和省、市委各项决策部署，突出聚力发展主题，把握稳中求进基调，激发改革创新动力，着力建设工业强市、优化生态环境、加快城乡一体、增进民生福祉、强化作风保障，以积极进取精神促进经济持续健康发展、社会和谐稳定。

　　建议今年全市经济社会发展主要预期目标为：地区生产总值增长8.5%以上；固定资产投资增长12%，力争增长15%；社会消费品零售总额增长12%；外贸进出口总额、出口额均增长7.5%；财政总收入、地方财政收入均增长8%；城镇居民人均可支配收入增长9.5%，农村居民人均纯收入增长9.5%以上；城镇新增就业4万人，登记失业率控制在4%以内；居民消费价格指数与省价格调控目标保持衔接；研究与试验发展经费支出占生产总值比例提高0.13个百分点；节能减排完成省下达任务。

　　按照上述总体要求和主要目标，经济社会发展要坚持稳中求进、改中求活、转中求好，政府工作要做到“五个坚定不移”：一是坚定不移抓改革、增活力。把改革创新贯穿于经济社会发展的各领域、各环节，突出政府自身改革先行、经济体制改革牵引，着力在要素配置市场化、城乡发展一体化等重点领域、关键环节的改革上取得突破，全面释放改革红利。二是坚定不移抓发展、强经济。深入实施工业强市、产业兴市战略，以提高经济增长质量和效益为中心，抓牢项目建设牛鼻子，着力扩投资、拓市场、兴实体，大力推进新型工业化、信息化、城镇化、农业现代化，努力做大经济总量、增强发展实力。三是坚定不移抓治水、促转型。牢固树立“绿水青山就是金山银山”发展理念，全力打好“五水共治”攻坚战，统筹治污水、防洪水、排涝水、保供水、抓节水，深入推进水气共治、城乡共治，全面改善生态环境，加快建设美丽湖州，倒逼经济转型升级。四是坚定不移抓实事、惠民生。抓住群众最关心、最直接、最现实的利益问题，一件接着一件办，一年接着一年干，努力实现城乡居民收入增长与经济发展同步，让改革发展的成果更多、更公平地惠及全体市民，保障群众安居乐业，确保社会安定和谐。五是坚定不移抓作风、优保障。坚持敢担当、重实干、善攻坚，以逢山开路、遇河搭桥的精神抓推进，以踏石留印、抓铁有痕的劲头抓落实，在干事创业中锤炼作风，在作风改进中保障发展，打造一流的政务环境，营造一流的发展氛围。

三、2014年重点工作

　　（一）以全面深化改革为统领，着力增强发展内生动力

　　加快政府职能转变和机构改革。深化简政放权，继续取消、下放一批市级行政审批事项。实施投资项目快速审批办法，抓好涉审中介服务机构清理规范工作，全面推进全流程高效审批服务。研究制定企业投资项目负面清单和市场准入标准，探索推进事前审批转向事后监管。推进政府性项目投融资体制改革，加强和规范债务管理。完成新一轮市、县区政府机构改革，优化机构设置、职能配置，加快推行权力清单制度。进一步推进事权下移、力量下沉，着力推动市本级加快发展、增强实力。

　　激发民营经济和开放型经济发展新活力。深入实施支持浙（湖）商创业创新、促进民营经济大发展大提升十大工程，鼓励民间资本办医、办学、办文化，参与基础设施建设，进入公用事业领域。继续推进“个转企、小升规、规改股、股上市”，引导民营企业加强制度创新、管理创新和商业模式创新，力争在多层次资本市场挂牌、上市企业15家。健全接沪融杭长效机制，积极对接和服务上海自贸区，借力发展新产业。打造通关、物流、保险、结汇、退税一站式外贸综合服务平台，构建外贸展会、跨境电商、境外设点、境外收购“四位一体”外贸发展新模式。深化区域合作，加强对口支援，推进对外交流。

　　推进资源要素市场化配置改革。统筹用地保障和耕地保护，新增建设用地指标1.8万亩，垦造耕地8000亩，完成农村土地综合整治复垦8000亩。推进工矿废弃地复垦利用工作，实施城镇低效用地再开发试点。探索建立农村集体经营性建设用地入市机制，全面推进农村宅基地确权登记发证，深化长兴农村宅基地用益物权保障制度改革试点。稳步调整城镇土地使用税政策，强化企业单位用地用电产出效益评价，加快健全用电、用水、用气阶梯价格、差别价格制度，完善排污权有偿使用和交易制度。全力争取扩大信贷有效投放，推进中小微企业、“三农”领域金融服务创新，力争金融机构贷款新增230亿元以上。深化地方金融改革创新，支持小额贷款公司等新型金融组织稳健发展，推动民间融资规范管理实现县区全覆盖。加快社会信用体系建设，维护区域信用环境和经济金融秩序。

　　（二）以提质增效为核心，着力加快经济转型升级

　　切实扩大有效投资。坚持引浙商、招央企、选外资多管齐下，着力招大、引强、选优，强化建链、延链、补链，完善“大好高”产业项目引进推进工作机制，力争省外浙商回归到位资金160亿元，央企合作实到资金30亿元，实到外资11.3亿美元。坚持引进市外项目和培育本土企业一视同仁，狠抓大项目建设，支持成长型企业，促进各类市场主体加大有效投入力度。围绕产业转型升级、城乡建设提升、公共服务完善、基础设施优化，实施市重点建设项目182项，力争当年完成投资500亿元。在工业六大重点特色产业和服务业领域，各实施百项重大项目，当年完成投资分别超百亿元。推动南太湖产业集聚区“一核四辅”高质量发展，做强核心区块、做优主导产业、做大优势企业。加快省际承接产业转移示范区开发建设，推动各类开发区、园区差异化竞争、特色化发展。

　　全面实施创新驱动发展战略。高标准推进国家创新型试点城市建设，进一步打造国家知识产权示范城市等四张科技名片。加快现代物流装备等3个省级高新技术产业园区以及22个省级以上高新技术特色产业基地建设，新认定国家重点扶持高新技术企业60家以上。深化与大院名校的政产学研合作，推广德清省级科技成果转化实验区建设经验，新引进重点创新载体15家，组织实施100项科技成果转化的产业化项目。深入实施南太湖系列人才引育工程，新增“国千”、“省千”人才15名左右。大力推进“四换三名”，淘汰涉及200家以上企业的落后产能，实施1000项左右的工业现代化技改项目，盘活存量建设用地6200亩，组织300家以上实体企业发展电子商务；推动企业兼并重组、做大做强，抓好质量强市建设和品牌创新、标准创新，实施领军型、成长型、创业型“三个一批”优秀企业家建设工程。

　　加快推动产业优化升级。大力推进工业创强，实施现代装备制造业产品创新、智能制造、品牌培育、技工培养“四位一体”提升行动计划，切实抓好现代物流装备等7个重点行业技术创新综合试点。全面完成印染、造纸、制革、化工四大行业整治提升年度任务，进一步加快吴兴织里童装、南浔小木业、德清小铸锻、长兴耐火、安吉小竹业等行业整治提升步伐。加快推进南太湖旅游综合体、天使乐园等重大旅游项目建设，推动南浔古镇争创5A级景区，启动建设湖州旅游公共服务（集散）中心，争创国家乡村度假（休闲）示范区。加快商业布局优化和流通网络建设，促进传统商贸业与现代网络经济融合发展。加快物流园区建设，推进安吉川达、南浔鑫达等重点内河支线项目。大力发展文化创意、服务外包、健康养生等新兴服务业。统筹粮食安全保障和品质农业发展，新建粮食生产功能区10万亩，全面完成11个省级现代农业综合区建设。加快建立现代种业发展体系，推广生态循环型农作新模式。大力培育家庭农场、农民合作社、农业龙头企业、新型职业农民，推进现代农业综合体建设，加快构建新型农业经营体系。

（三）以“五水共治”为突破口，着力推进生态文明建设

　　全面加强环境综合治理。坚持“五水共治、治污先行”，认真落实四级“河长制”和河流交界断面水质考核奖惩制度，加快城镇污水处理厂提标改造和污水处理配套管网建设，农村生活污水治理设施确保实现行政村全覆盖、尽快实现自然村全覆盖，年内覆盖到70%的规划保留自然村，农户受益率提高到50%以上，新建设施运行负荷率达到70%以上，确保取得水环境“中期清”的阶段性成果。大规模开展河道清淤整治，加快农田水利五项标准化工程建设，不断提高防洪抗洪能力；加强城镇排水管网和泵站建设，切实保障城镇汛期安全；强化水源地保护，加快城乡一体供水设施建设，确保城乡居民饮用水安全；严格执行水资源开发利用控制、用水效率控制和水功能区限制纳污“三条红线”，积极推进节水型城市建设。以治尘、治烟等为重点，进一步加大雾霾治理力度，深化大气复合污染综合防治，加强道路扬尘、露天堆场、秸秆焚烧等治理工作，加快工业废气、车船尾气等治理进度，有效改善环境空气质量。推进废旧物资回收利用体系建设，开展污泥刷卡转运工作试点，建立土壤污染联防机制。坚持减点控量、治污达标，继续强化矿山企业综合治理。深入推进“四边三化”，巩固整治成果，完善长效机制。继续推进“三改一拆”，全面开展无违建县区创建工作，着力加强拆后利用和建设。

　　切实抓好节能减排和生态保护。严格实行新建项目能耗等量置换、减量置换制度，健全空间、总量、项目“三位一体”环境准入体系，从源头上控制和减少高能耗、重污染项目建设。组织实施节能技改“双百”行动和60项以上污染减排重点项目，加大煤改气、光伏发电、光热利用推广力度，加快工业企业、规模畜禽养殖场治污设施建设与改造提升。开展企业核定用能工作，加强公共机构节能管理，推进建筑、交通、商贸等领域的节能降耗。加强排污企业规范化管理，推进刷卡排污系统建设。全力创建国家生态文明建设先行示范区，完成环境功能区划编制，加快全国水生态文明城市建设试点，推进林业碳汇试验和试点，逐步健全生态补偿机制。加快生态县区、生态市联创步伐，力争通过省级生态市验收。

（四）以城乡一体化为目标，着力提升新型城市化水平

　　统筹中心城市和小城镇建设。加快推进龙溪港东岸改造、小西街历史文化街区修缮整治等一批重点项目，不断提升湖州中心城市核心区功能。加快综合交通枢纽区块、黄芝山西拓区、康山分区等开发建设，打造湖州开发区发展新空间。完成南太湖区域概念性规划编制，加快启动长兜港以东片区基础设施建设，推进南太湖一体化开发。启动梁希森林公园二期、湖山大道项目，稳步推进南郊风景区开发建设。加大对吴兴、南浔城市建设的支持力度，切实提升城市整体发展功能。基本建成中心城市外环线项目，加快城市内部交通微循环建设，大力发展公共交通，进一步缓解城市交通拥堵。推进智慧城市建设，构建“大城管”格局，提高城市精细化管理水平。全力抓好德清省级城乡体制改革试点，推动三县城市建设不断跃上新水平，促进县城与湖州中心城市联动发展。启动小城市培育试点新三年行动计划，加快中心镇改革发展，选择有条件的地方开展新型城镇化、城乡一体化先行先试。

　　打造美丽乡村升级版。深化美丽县区、乡镇、村、户“四级联创”，提升和建成5条美丽乡村示范带。加强历史文化村落保护和利用，加快中心村培育和建设，打造一批精品村、特色村。深化农村环境连片整治，完善“户集、村收、乡镇运、市县处理”的农村垃圾收集处置模式，探索构建城乡一体环境保洁长效管理机制。全面开展市区美丽乡村路建设行动，全市建成农村联网公路200公里，完成农村公路大中修230公里，加固和改造病害桥梁60座。大力拓宽村级集体经济发展途径，加强农村集体资金、资产、资源阳光化管理，发展农村产权流转交易市场。深入推进八里店南片新农村综合改革试验区和南浔城南城乡一体化发展试验区建设。深化与浙江大学的市校合作，拓宽合作领域，提升合作层次。

　　加快重大基础设施建设。完成杭宁高速公路拓宽项目前期，加快杭长高速公路北延、 318国道改线工程建设，建成104国道长兴夹浦段等干线公路项目，启动实施京杭运河湖州段航道整治工程。加快推进商合杭铁路、湖苏沪铁路、申嘉湖高速公路西延项目前期，继续深化轨道交通规划研究。加强太湖流域水环境综合治理，全面推进四大重点水利工程建设。不断加强电力、信息化等基础设施建设，提升安全保障和高效运营水平。

　　（五）以民生改善为根本，着力促进社会和谐稳定

　　加快民生事业发展。千方百计增加城乡居民收入，做好低收入群众增收工作。加大对“零就业”家庭、失业人员、被征地农民等重点人群就业帮扶力度，做好高校毕业生就业引导和创业扶持工作。引导适合人群积极参加社会保险，稳步提高参保人员待遇水平。整合市区新农合制度，推进城乡居民基本医疗保障一体化。不断健全社会养老服务体系，统筹推进社会救助体系建设。支持慈善等公益事业健康发展。启动第二轮学前教育发展行动计划，推进义务教育优质均衡发展，促进高中教育特色多样发展，加快现代职业教育体系建设，提升高等教育发展水平。加快文化基础设施和基层网络建设，打造“文化街景”等特色品牌，加大文艺精品创作支持力度。进一步加强历史文化名城和文化遗产保护工作。不断完善公共体育设施，推进全民健身，发展竞技体育。实施市级公立医院改革，优化和调整医疗资源布局，加强中心城区社区卫生服务体系和基层医疗卫生机构能力建设，推进和规范医师多点执业制度，加快创建省级卫生强市。启动市中心医院迁建项目前期工作，加快吴兴区人民医院、南浔区人民医院建设。落实“单独两孩”政策。稳步推进户籍管理制度改革，促进基本公共服务均等化。加大政府购买公共服务力度，促进社会力量参与社会事业建设。

　　推进社会治理创新。坚持系统、依法、综合、源头治理，大力提升“平安湖州”建设水平。分类推进乡镇、街道社会服务管理中心规范化建设，促进基层公共服务、社会治理水平的提升。实行四类社会组织直接登记制度，推动社会组织更好发挥作用。加快城乡社区建设，推进基层民主自治。培育法治文化，加强法律服务，推动涉法涉诉信访纳入法治轨道解决。完善民意导向型警务新模式，深化社会治安防控体系建设。健全流动人口、特殊人群服务管理体系。不断推进安全生产监管机制创新，加大安全隐患排查和整治力度，有效防范和坚决遏制安全生产事故。完善食品药品全过程监管和可追溯制度，提高监管效率和水平。推进应急联动体系建设，提高气象、地质等灾害防御能力。加强国防动员和人民防空建设，推动军民融合深度发展。

　　各位代表！解决民生问题是最大的政治，改善民生福祉是最大的政绩。今年，我们将全力以赴办好以下十方面民生实事项目：一是城乡治水。启动建设太湖水厂。开工建设苕溪防洪与清水入湖河道整治工程。完成170公里以上城镇污水配套管网建设。建筑石料实际开采量控制在5000万吨左右，在产矿山100%达到市级以上绿色矿山标准。督促66家造纸、印染行业重点涉水企业完成整治提升。完成中心城区二环西路（江南车城）、太湖路－陵阳路口、外环东路－苕溪路口、青铜路－龙王山路口4个内涝点的治理。二是大气治理。督促浙能长兴电厂完成1台火电机组脱硝改造，统一能源热电、织里长和热电、南太湖热电、协鑫热电、加怡新市热电等企业完成8台热电机组脱硫改造，湖州南方水泥集团完成4条水泥熟料生产线脱硝改造。创建建筑施工场所绿色工地30个。实施中心城区过境渣土、矿石运输车辆加盖密闭全天候监管。中心城区高污染机动车限行区域扩大10平方公里以上。中心城区城市道路机械化清扫率达到50%。三是食品安全。新增无公害农产品、绿色食品、有机食品“三品”生产基地6万亩。完成10家农贸市场改造提升；创建流通环节食品安全电子化管理示范单位50家。食品生产企业抽检覆盖率达到100%。四是住房保障。新竣工保障性住房5356套。对950户农村困难群众危旧房实施改造。建成渔民陆上安置房950套。五是宜居环境。创建市级美丽乡村40个。创建市级生态村50个。完成中心城区10个老小区综合整治。六是公共交通。新增、更新公交车100辆，调整和优化公交线路20条。完成中心城区三环东路－吴兴大道、二环西路－西塞山路等5个拥堵点的治理；打通高富路延伸、所前街延伸等4条断头路；建成时代广场公交换乘中心和馨水园、太湖度假区公交首末站；新增公共自行车2000辆。七是就业帮扶。帮扶困难人员就业3000人。开展残疾人就业帮扶2000人。八是医疗服务。完成湖州健康信息服务平台建设，全市医院实现检查检验报告网上查询，市级医院实现分时段预约挂号；完善基层“20分钟医疗卫生服务圈”，新（迁）建和改扩建社区卫生服务中心12家、社区卫生服务站98家；实施尿毒症患者血透服务提升工程，增强市中心医院、市中医院、市第一医院以及县区医院血透服务能力，新增血透机60台。完成红十字应急救护普及培训4.5万人以上。九是养老助残。新建成居家养老服务照料中心200家；对1万名生活困难老人给予养老服务补贴。对计生失独父母给予特扶金和社会养老保险政府补助。为4万名残疾人购买意外伤害保险，为500户困难残疾人家庭免费改造无障碍设施。十是教育文体。中等职业教育学生学费减免惠及6万人次，“营养改善计划”惠及义务教育段家庭贫困学生2万人次，助学券惠及家庭困难中小学生2000人次。建成农村文化礼堂100个。开展文化走亲200场、中心城区社区数字电影放映200场。新建成市级社区全民健身活动示范点50个。

　　（六）以开展党的群众路线教育实践活动为契机，着力加强政府自身建设

　　努力建设法治型政府。全面贯彻浙江省法治政府建设实施标准，把依法行政的各项要求落实到政府工作的各个方面。自觉接受人大依法监督、政协民主监督，坚持重大决策出台前向人大报告、听取政协意见，认真执行人大及其常委会决议、决定，高质量办理人大代表建议、政协提案。主动接受舆论监督和社会公众监督，完善重大事项集体决策、专家咨询、公众听证、社会公示制度。推进“湖州发布”政务微博建设，提升政府信息公开水平。全面推行行政执法责任制，促进规范执法、公正执法、文明执法。

　　加快建设服务型政府。扎实开展党的群众路线教育实践活动，严肃整治“四风”突出问题，建立健全密切联系群众、切实改进作风的常态化制度、长效化机制。深化“两看两比”活动，全面加强以提高行政效能为重点的政府执行力建设，大力推进政府工作项目化管理、责任化考核，促进政府服务提速、提质、提效。完善“12345政府阳光热线”运行机制，着力提高群众满意度。切实加强教育、监督、管理，努力建设一支讲政治、顾大局、有激情、能干事的高素质公务员队伍。

　　全力建设廉洁型政府。坚持用制度管权、管事、管人，扎实推进惩治和预防腐败体系建设，认真落实廉政建设责任制，严格执行各项纪律规定，努力做到干部清正、政府清廉、政治清明。厉行勤俭节约，严禁铺张浪费，“三公”经费预算总额在上年预算基础上压缩30%。加强财政资金和政府性投资项目监督管理，切实提高资金使用绩效。坚决纠正损害群众利益的不正之风，严肃查处各类违纪违法案件。

　　各位代表！发展成就令人鼓舞，改革势头催人奋进。面对新形势、新任务、新要求，让我们在中共湖州市委的领导下，凝聚全市人民的智慧和力量，解放思想、振奋精神，敢于担当、真抓实干，为实现“四个翻番”、打造“四个湖州”，加快建设现代化生态型滨湖大城市而努力奋斗！
